# Development and usability testing of a fully immersive VR simulation for REBOA training

**DOI:** 10.1186/s12245-023-00545-6

**Published:** 2023-10-06

**Authors:** T. Birrenbach, R. Wespi, W. E. Hautz, J. Berger, P. R. Schwab, G. Papagiannakis, A. K. Exadaktylos, T. C. Sauter

**Affiliations:** 1 Department of Emergency Medicine, Inselspital, University Hospital Bern, Freiburgstrasse 16C, Bern, CH-3010 Switzerland; 2 Schutz und Rettung Bern, Sanitätspolizei Bern, Bern, Switzerland; 3 ORamaVR SA, Geneva, Switzerland; 4 Institute of Computer Science, Foundation for Research and Technology, Hellas, Heraklion, Greece; 5 Department of Computer Science, University of Crete, Heraklion, Greece

**Keywords:** REBOA, Virtual reality, Trauma resuscitation, Medical education

## Abstract

**Background:**

Resuscitative endovascular balloon occlusion of the aorta (REBOA) is a potentially life-saving procedure for bleeding trauma patients. Being a rare and complex procedure performed in extreme situations, repetitive training of REBOA teams is critical. Evidence-based guidelines on how to train REBOA are missing, although simulation-based training has been shown to be effective but can be costly and complex. We aimed to determine the feasibility and acceptance of REBOA training using a fully immersive virtual reality (VR) REBOA simulation, as well as assess the confidence in conducting the REBOA procedure before and after the training.

**Methods:**

Prospective feasibility pilot study of prehospital emergency physicians and paramedics in Bern, Switzerland, from November 2020 until March 2021. Baseline characteristics of trainees, prior training and experience in REBOA and with VR, variables of media use (usability: system usability scale, immersion/presence: Slater-Usoh-Steed, workload: NASA-TLX, user satisfaction: USEQ) as well as confidence prior and after VR training were accessed.

**Results:**

REBOA training in VR was found to be feasible without relevant VR-specific side-effects. Usability (SUS median 77.5, IQR 71.3–85) and sense of presence and immersion (Slater-Usoh-Steed median 4.8, IQR 3.8–5.5) were good, the workload without under-nor overstraining (NASA-TLX median 39, IQR 32.8–50.2) and user satisfaction high (USEQ median 26, IQR 23–29). Confidence of trainees in conducting REBOA increased significantly after training (*p* < 0.001).

**Conclusions:**

Procedural training of the REBOA procedure in immersive virtual reality is possible with a good acceptance and high usability. REBOA VR training can be an important part of a training curriculum, with the virtual reality-specific advantages of a time- and instructor-independent learning.

**Supplementary Information:**

The online version contains supplementary material available at 10.1186/s12245-023-00545-6.

## Background

Trauma is the leading cause of death in patients under 45 years of age [1]. In particular, non-compressible torso hemorrhage resulting in hemorrhagic shock bears a high mortality and morbidity [2–4]. Resuscitative endovascular balloon occlusion of the aorta (REBOA) has recently gained popularity as a potentially life-saving procedure, by allowing quick transitory hemorrhage control for truncal injuries [5–9]. A recent meta-analysis suggests a positive effect of REBOA in non-compressible torso injuries when compared to resuscitative thoracotomy [10], as does a recent propensity score-matched analysis [11]. Different international guidelines on polytrauma management suggest its application in unstable trauma patients who are unresponsive to other resuscitative efforts [12–14].

The application of REBOA is also under investigation during cardiopulmonary resuscitation, as it increases coronary and cerebral perfusion pressure [15, 16].

However, because REBOA is a rare and complex procedure performed in extreme situations with the potential to cause great harm to the patient, mastery of this particular skill is critical. High-volume deployment centers show increased survival of REBOA patients when compared to low-utilization centers [17].

REBOA training is usually done by simulation or a combination of simulation with knowledge transfer done with lectures or/and eLearning. Although a recent systematic review confirms the effectiveness of simulation-based training, there is still confusion about optimal course design, effect size, skill transfer, and skill retention [18], and evidence-based guidelines on how to train REBOA are missing. Simulation training is usually done with a high-fidelity endovascular simulator (e.g., Mentice VIST, Mentice, Gothenburg, Sweden) or live animal models [18]. Traditional simulation-based training therefore is very resource intensive, including high costs for training materials (e.g., high-fidelity manikin, REBOA training catheters) and personnel resources.

Virtual reality (VR) is a technology that immerses the user in an artificial 3D environment with the use of a head-mounted device (VR headset). Interaction with the virtual environment takes place via wearable devices (controllers) or even with the user’s own hand (hand tracking). VR simulations have proven to be a useful and effective tool, mainly for training surgical and technical skills [19–22]. VR simulation training offers a scalable, autonomous (time- and location-independent) experience, especially for settings that prove to be too risky or resource-intense for traditional simulations.

To our knowledge, there is no fully immersive virtual reality simulation for REBOA training so far.

At the “Schutz und Rettung Bern” [23], we recently started a clinical trial on REBOA in non-traumatic cardiac out-of-hospital cardiac arrest [24]. While the actual insertion of the REBOA catheter in this setting is performed by a core team of 4–5 senior emergency physicians, we need to train the regular prehospital teams (prehospital emergency physicians and emergency paramedics) in the basic principles of the REBOA procedure so that they understand the procedure and can assist the core REBOA team. We thus aimed to.

i) Develop a fully immersive VR REBOA training simulation
ii) Determine the feasibility of the application of the VR REBOA simulation at the local prehospital emergency medical services
iii) Evaluate the acceptance of the VR REBOA simulation (usability, simulator sickness, sense of presence and immersion, workload, user satisfaction)
iv) Examine the subjective confidence in conducting the REBOA procedure before and after the simulation training.

## Methods

### Development of the fully immersive VR simulation

To realistically recreate a medical procedure in VR, we have to understand its basic steps, actions, and milestones. The best way to obtain such a breakdown analysis is to consult professionals specialized in this method. In our case, the Subject Matter Experts material, required for the design of a complete storyboard tailored for the REBOA VR training module, was provided by our medical experts (TB, WEH, TCS) to the development team of ORamaVR (Geneva, Switzerland), according to the methodology published in [25].

The simulated environment consisted of an emergency theatre including a virtual patient, who is hemodynamically unstable after a motor vehicle accident with free fluid in the abdomen. Clinical information, vital signs, ultrasound, or X-ray images, as well as information on the next steps are displayed on monitors in the virtual emergency room. A sterile covered table is used to store and prepare the required materials. The insertion of the REBOA catheter is performed step by step on a virtual person. These steps include.

1. Decision on placement depth and measurement for zone 1 REBOA using the clinical information
2. Preparation of femoral arterial access
3. Cannulation of the common femoral artery using ultrasound
4. Guidewire introduction
5. Placement of sheath
6. REBOA catheter preparation
7. REBOA catheter insertion
8. Balloon inflation and confirmation of its effect
9. Fixation of catheter
10. Chest X-ray

**Fig. 1:**
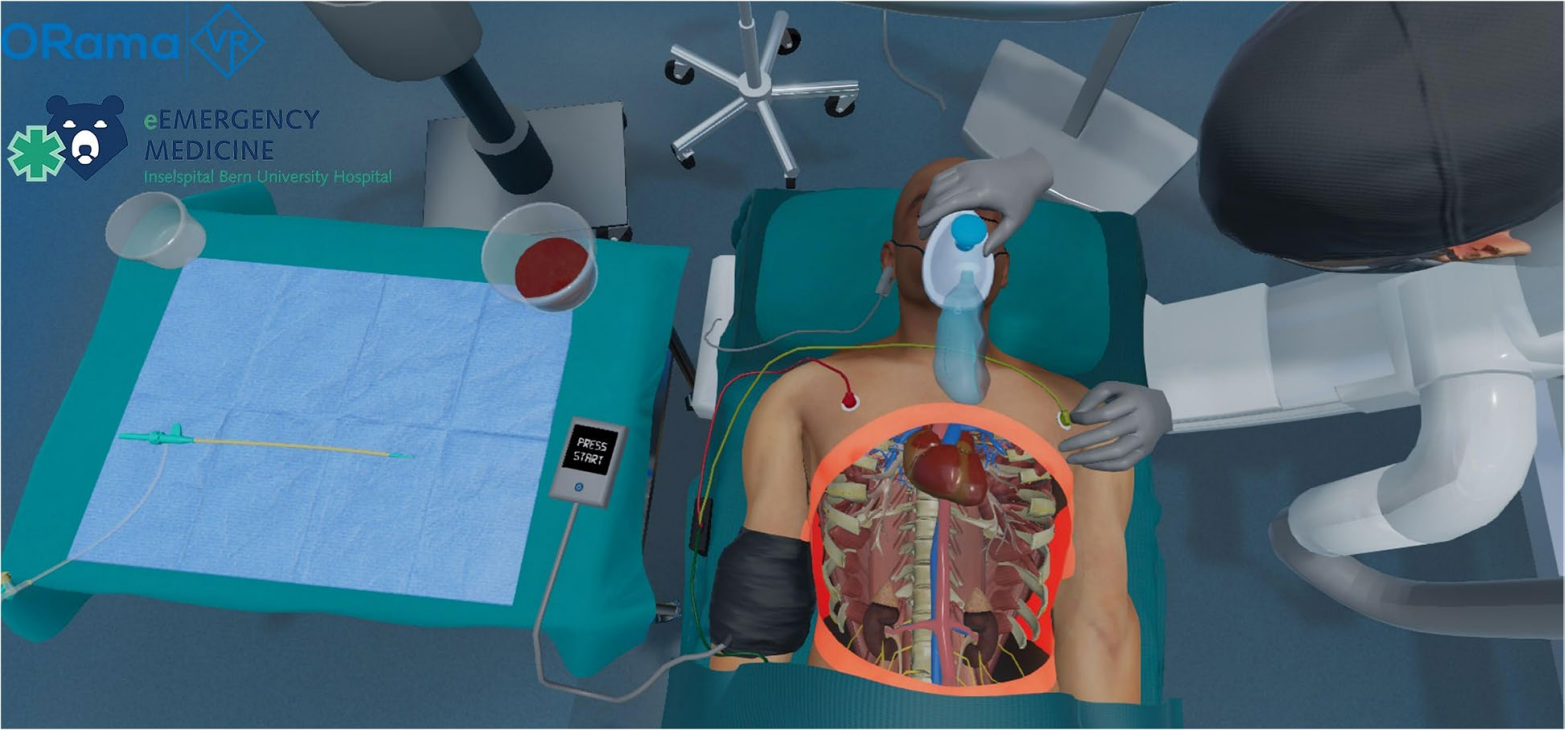
Screenshot of the VR REBOA simulation. Preparation of the sheath, adaptable view into the torso

**Fig. 2:**
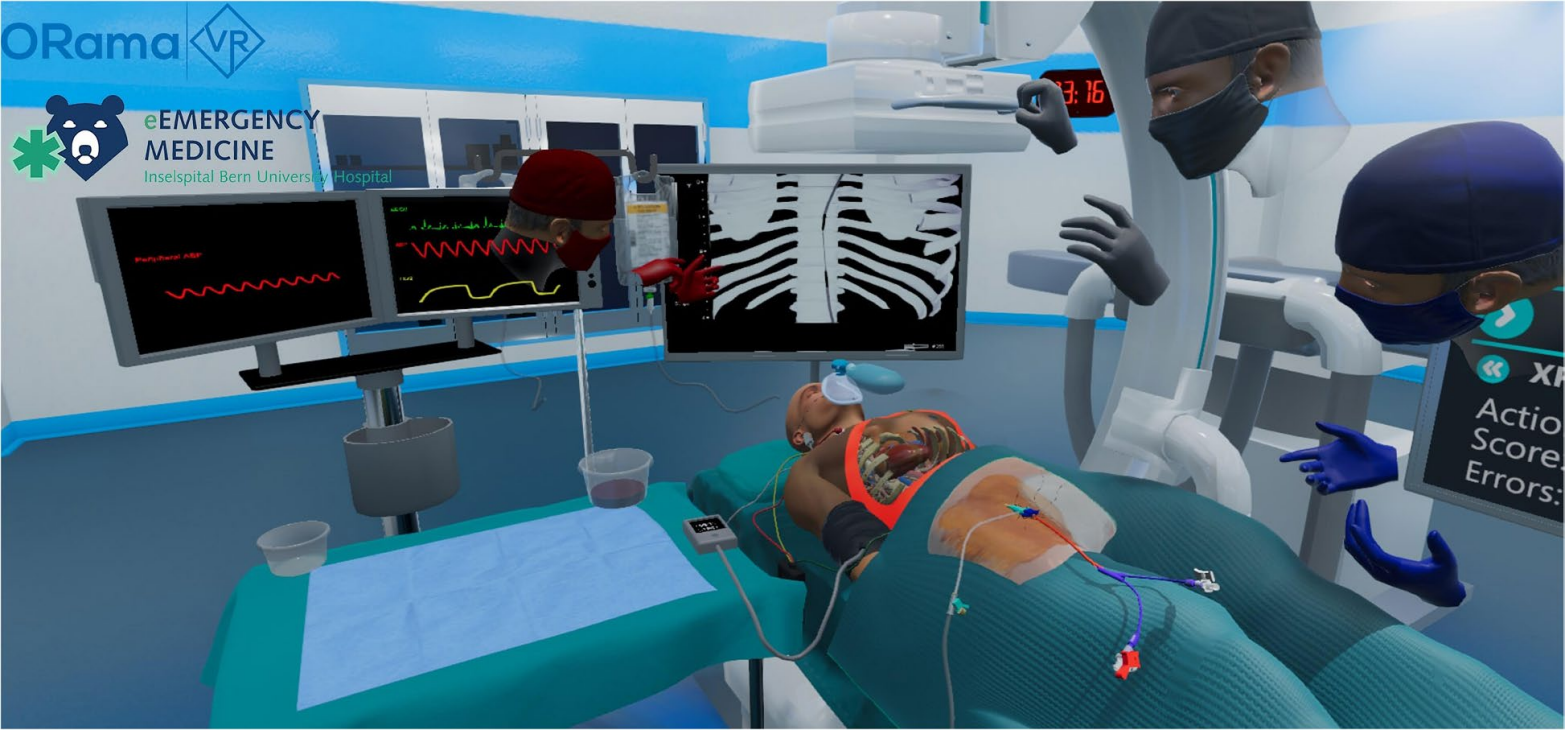
Screenshot of the VR REBOA simulation. Patient monitorized in the resuscitation bay, REBOA catheter in situ after successful insertion

Screenshots of the simulation are detailed in Figs. 1 and 2.

Two modes of action were available for single-player use: In the tutorial mode visual aids and prompts helped the trainees in providing information on the next procedural step; these prompts were missing in the normal mode.

### Study design, setting, and ethical approval

This is a prospective feasibility pilot study involving prehospital emergency physicians and emergency paramedics of the “Schutz und Rettung Bern” [23]. The study was conducted at the University Emergency Department (Universitätsklinik für Notfallmedizin) at the Inselspital, University Hospital, Bern, Switzerland. The study took place from November 2020 until March 2021.

This study was exempt from full ethical review by the local institutional review board (Kantonale Ethikkommission Bern (Ethics Committee Bern), BASEC-No: Req-2020-00970). Written informed consent for study participation was obtained from all participants. Written informed consent from a parent and/or legal guardian is “not applicable”. Consequently, the present study was conducted in accordance with the ethical standards of the 1964 Declaration of Helsinki and its subsequent amendments.

### Participants

The local prehospital emergency medical services (“Schutz und Rettung Bern”) are carrying out about 23,000 preclinical medical emergency operations annually. There are 17 emergency physicians and 108 paramedics (50% female) working in a rendezvous system. All participants were offered and attended the training on a voluntary basis and we provided no remuneration. Written informed consent was obtained for the study and publication of the study results. Written informed consent from a parent and/or legal guardian is “not applicable”.

### Baseline data

Sociodemographic data (gender, age, profession (physician/paramedic), working experience in years, need to wear eyeglasses, right/left-handedness), prior training and experience in REBOA as well as prior experience with VR, were collected in a survey.

### Intervention

**Fig. 3:**
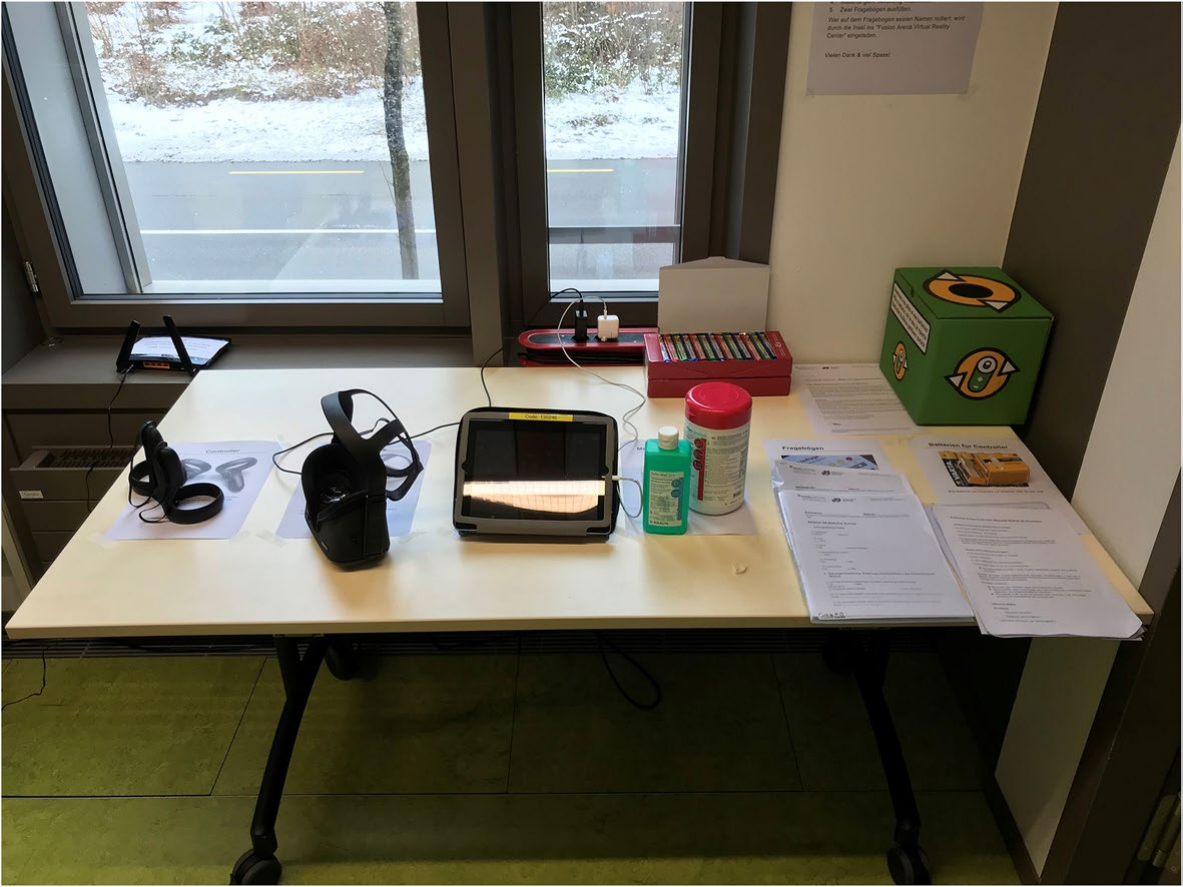
Setup of the VR simulation. Setup for the VR REBOA simulation including hand-held controllers, head-mounted device (Oculus Quest), tablet PC, disinfection materials

Initially, three peer teachers were introduced to the VR set-up and the correct operation of the REBOA VR module by the development and study team of the University Emergency Department (TS, TB, JB) in a 2-h training session, who then passed on their knowledge and were able to train their peers (“teach the teacher”). The REBOA VR simulation station was set up in an empty room at the Schutz und Rettung headquarter Bern (Fig. 3). The hardware consisted of a stand-alone VR headset with two hand-held controllers (Oculus Quest, Oculus VR, Facebook Inc., Menlo Park, CA, USA) and a tablet pc. The REBOA module, version 1.2.6, software platform, developed by ORamaVR (Geneva, Switzerland), was used in the single-player tutorial and normal mode.

During 5 months, from November 2020 to March 2021, paramedics and preclinical emergency physicians had the opportunity to train with the VR simulation on their own after being instructed by the trained peer instructors during their shifts or whenever was a suitable timeslot for them. The peer instructors had the opportunity to follow the simulation on the tablet PC, and thus to provide additional targeted assistance, apart from the automated feedback by the tutorial mode of the software, if necessary.

### Outcome measures

#### Acceptance of the VR simulation

Evaluation of acceptance of the VR simulation was carried out according to established questionnaires directly after the VR training.

#### Usability

Usability was assessed using the System Usability Scale (SUS) [26], which is composed of 10 questions with a 5-point Likert attitude scale and the After-Scenario Questionnaire (ASQ) [27], which assesses the ease of task completion, satisfaction with completion time and satisfaction with supporting information on a 7-point Likert scale (total score ranges from 1 = full satisfaction to 7 = poor satisfaction).

#### Simulator sickness

“Visually-induced motion sickness” was assessed with four items (The VR training caused nausea/headache/blurred vision/dizziness) taken from the Simulator Sickness Questionnaire (SSQ) from Kennedy et al. (Likert scale from 1 = totally disagree to 5 = totally agree) [28].

#### Sense of presence and immersion

Presence and immersion in the virtual world were determined according to the 6-item questionnaire developed by Slater-Usoh-Steed (total score ranges from 1 = no immersion to 7 = full immersion) [29].

#### Workload

Perceived subjective workload on a scale from 0 to 100 was assessed using the NASA-Task Load Index (NASA-TLX) as a total score and within 6 subdomains [30]. Overstraining is associated with a total score > 60, and understraining with a total score of < 37 [31].

#### User satisfaction

The User Satisfaction Evaluation Questionnaire (USEQ) has six questions with a 5-point Likert scale to evaluate user satisfaction (total score ranges from 6 = poor satisfaction to 30 = excellent satisfaction) [32].

Furthermore, free-text comments were collected.

#### Subjective effectiveness/confidence

Confidence in the correct performance of the REBOA intervention was assessed before and after the training (“I feel confident in conducting the REBOA intervention correctly” (Likert scale from 1 = totally disagree to 5 = totally agree).

### Statistical analysis

Data was analyzed using SPSS.

Baseline characteristics are presented as numbers and percentage or median and interquartile range (IQR) using descriptive statistics as appropriate. Pre- and post-simulation comparisons were performed with the Wilcoxon signed rank test. A *p* value < 0.05 was considered significant.

## Results

### Development of the VR REBOA simulation

To create the VR training, we used MAGES 4.0, which enables rapid prototyping of shared, collaborative networked medical training in VR [32].

### Feasibility

VR REBOA training for paramedics and emergency physicians was found to be feasible. The chosen peer teaching format was well accepted and implemented by the participants and confirmed as a very useful approach. The use of the VR simulation in only one empty room without further equipment was possible and enabled spontaneous practice sessions without scheduled training hours or permanently reserved training rooms and personnel (Fig. 3).

### Baseline characteristics of the study population

Baseline characteristics of the study population are detailed in Table 1. Of the 45 participants, 4 (8.9%) were physicians. None of the participants had received prior training in REBOA or had ever carried out the procedure in real life. Participating physicians had limited experience in femoral arterial cannulation. The majority of the participants did not regularly use of video games or VR.

**Table 1:** Baseline characteristics of the study population (*n* = 45)

**Sociodemograhic factors**	
Gender, female (yes), [*n* (%)]	21 (46.7)
Age, [median (IQR)]	34 (30.5–40)
Profession, [*n* (%)]	
Physician	4 (8.9)
Paramedic	41 (91.1)
Working experience in years, [median (IQR)]	8 (6–14.5)
Glasses (yes), [*n* (%)]	10 (22.2)
Right-handedness (yes), [*n* (%)]	40 (88.9)
**Prior experience in REBOA**	
Prior training in REBOA, (yes), [*n* (%)]	0 (0)
Prior REBOA insertion, (yes), [*n* (%)]	0 (0)
No. of femoral arterial cannulations (physicians only,* n* = 4), [n (%)]	
10	2 (50)
20	1 (25)
30	1 (25)
**Prior experience in VR, [*n* (%)]**	
“I play computer games regularly”, Likert Scale 1–5	
1 totally disagree	28 (62.2)
2	4 (8.9)
3	4 (8.9)
4	6 (13.3)
5 totally agree	3 (6.7)
“I regularly use VR”, Likert Scale 1–5	
1 totally disagree	39 (86.7)
2	6 (13.3)
3	0 (0)
4	0 (0)
5 totally agree	0 (0)

*Abbreviations*: *IQR* interquartile range, *no* number, *REBOA* resuscitative endovascular balloon occlusion of the aorta, *VR* virtual reality

### Acceptance of the VR REBOA simulation (usability)

Overall, the VR REBOA simulation was well received by the participants (Table 2). Usability measured with the SUS was clearly above the average of 68 (median 77.5, IQR 71.3–85), indicating good usability. The simulation was very well tolerated. Sense of presence and immersion according to Slater-Usoh-Steed was good (median 4.8, IQR 3.8–5.5). Workload as measured in the NASA-TLX was in the desired range (neither under- nor overstraining with a median of 39, IQR 32.8–50.2). User satisfaction measured by the USEQ scored a median of 26 of 30 points (IQR 23–29).

**Table 2:** Acceptance of the VR REBOA simulation

**Usability**	
System Usability Scale (SUS), [median (IQR)] Range 0–100	77.5 (71.3–85)
**Satisfaction, [median (IQR)]**	
User Satisfaction Evaluation Questionnaire (USEQ), [median (IQR)] Range from 6 = poor satisfaction to 30 = excellent satisfaction	26 (23–29)
After-Scenario Questionnaire (ASQ), [median (IQR)] Range from 1 = full satisfaction to 7 = poor satisfaction	2 (1.7–2.8)
**Simulator sickness, [median (IQR)]** Likert Scale 1–5 (1 = no symptoms)	
Nausea	1 (1–2.5)
Headache	1 (1–1)
Blurred vision	1 (1–1.5)
Dizziness	1 (1–2)
**Sense of presence and immersion**	
Presence and Immersion according to Slater-Usoh-Steed, [median (IQR)] Range 1–7 (7 = full presence and immersion)	4.8 (3.8–5.5)
**Workload, [median (IQR)]**	
NASA-TLX (total score)	39 (32.8–50.2)
Mental demand	125 (90–250)
Physical demand	30 (12.5–70)
Temporal demand	45 (22.5–95)
Performance	225 (135–300)
Effort	55 (17.5–77.5)
Frustration	15 (0–55)

*Abbreviations*: *ASQ* After-Scenario Questionnaire, *IQR* interquartile range, *NASA-TLX* NASA-Task Load Index, *SUS* System Usability Scale, *USEQ* User Satisfaction Evaluation Questionnaire

Free-text comments of the participants generally indicated a good acceptance (e.g., “Great way to practice courses of action or scenarios”; “I thought the VR experience was great and was able to get a good look into the REBOA catheter procedure”). However, critical aspects were illuminated as well (e.g. “Interesting experience to have a VR headset on my head for once. But for me this is no substitute for other means of education, as there is too much support needed. Cost/benefit ratio is not right for me.”). The complete free-text comments of the participants are detailed in Supplement 1.

### Confidence

Subjective confidence of the participants in using the REBOA procedure correctly before training was low and significantly increased after the VR simulation (Table 3).

**Table 3:** Confidence

**Confidence**	**Pre**	**Post**	***P***
“I feel confident conducting REBOA correctly”, Likert Scale 1–5, [*n* (%)]			0.0001
1 totally disagree	42 (93.3)	24 (53.3)	
2	1 (2.2)	9 (20)	
3	1 (2.2)	8 (17.8)	
4	0 (0)	3 (6.7)	
5 totally agree	1 (2.2)	1 (2.2)	

*Abbreviation*: *REBOA* resuscitative endovascular balloon occlusion of the aorta

## Discussion

### Summary

Training with a fully immersive VR simulation for REBOA is feasible with a good usability, high satisfaction, and optimal workload during training. We showed that the VR training increased familiarity with the procedure with little VR training-associated side effects.

### Feasibility

Our study shows that it is possible to set up and conduct REBOA VR training on a population without prior knowledge of the procedure and in any given location without specific preparations necessary. Compared to a traditional simulation center, this type of implementation does not require any special constructional prerequisites but only an empty playing area.

The study participants found their way easily within the simulation and were able to run through it independently after a brief introduction, although they had no previous experience with VR or other computer games. Since the simulation has a tutorial mode and a realistic game character, the use was easy and intuitive for the majority of the test subjects. Some participants initially needed help from a peer teacher outside of the simulation, which could be provided through observation on an adjunct tablet computer. Since the VR training is an autonomous gaming experience, it does not necessarily require a trained instructor with specific medical knowledge. The peer instructor concept is based on the “teach the teacher” principle and can thus save expensive human resources as well as instructor time. As the system shows and constantly controls the correct execution and sequence of the skills to be learned, the VR simulation can be used time and instructor-independent.

### Acceptance

The usability, measured with the System Usability Scale (SUS), was high. To evaluate user satisfaction, the key component of usability from different perspectives, we confirmed good user satisfaction with both ASQ and USEQ. When evaluating a new training method such as VR, it must be taken into account that the evaluation may be subject to the novelty effect, and the usability measurement results may be overestimated [33].

In addition to high satisfaction and good usability in general, participants indicated they experienced a high level of presence and immersion in the VR training without significant side effects. This high level of immersion and presence could be achieved through immersive VR technology with head-mounted displays, which, to the best of our knowledge, we are investigating for the first time for a REBOA training, and by avoiding immersion-interrupting elements, e.g., dialog boxes or drop-down menus whenever possible. It has been argued that a high level of presence and immersion in VR can be an indicator of cognitive engagement with the content of the virtual environment, and thus an important predictor of experiential learning [34, 35].

### Confidence

Although the confidence after the VR training had increased significantly in the pre-post-comparison, a relevant number of participants still reported a low confidence.

Since VR training is limited in terms of haptic experience and knowledge transfer is also easier to teach using classic learning methods, such as self-study e-learning, it is recommended to integrate a VR training into a dedicated learning curriculum. VR training is not intended to replace any other training but to supplement it. An example is the out-of-hospital-cardiac-arrest training curriculum by Brede et al. [36]. However, this problem is not specific to our REBOA training setting shown here but has already been demonstrated in other settings. Since our goal was not to train and enable our participants to self-administer the REBOA catheter in the planned clinical trial, we simply wanted to improve their knowledge and understanding of the procedure and enable them to support the REBOA core team in the field.

Another possible application of VR training could be self-guided training to prevent skill decay, a well-known and relevant problem in the teaching of skills [37]. Park et al. showed REBOA skill degradation was most pronounced in surgical trainees who did not receive training for more than 5 months [38]. There are no studies to date on the best way to teach REBOA in the long term and how to minimize skill decay. However, the persistence of knowledge learned in VR over one month was previously demonstrated for procedural teaching [35]. Given the time-, location-, and instructor-independent nature of our REBOA training, it could be an ideal way to support regular self-guided repetition training to prevent skill decay. Further long-term research is needed.

## Limitations

These results must be interpreted with some limitations. First, this was a study of a single population with a limited number of participants who may be subject to selection bias due to voluntary participation, thus impacting generalizability. Although technically possible in the present VR simulation, in our study setting the training was conducted by only one person at a time. This lacks the opportunity to train teamwork skills that are essential for effective work in emergency settings. However, procedural skills are the basis of any teamwork and the present simulator was designed to teach these procedural basics. Likewise, a non-haptic VR simulation cannot be a substitute for haptic skills training such as sonography-guided vascular puncture, underscoring the use of VR simulation as a supplement to, rather than a substitute for, a REBOA training curriculum.

In our study, only a subjective measure of the effectiveness of the training was conducted using self-rated confidence pre- and post-training.

Due to the infrequency of the trained procedure, objective outcomes at the patient level are difficult to collect. One potential approach for future studies could be the use of the REBOA rate instrument [39].

## Conclusion

The procedural training of the REBOA catheter procedure in immersive virtual reality is possible with a good acceptance and high usability indicated by the trainees. REBOA VR training can be an important part of a training curriculum, with the virtual reality-specific advantages of a time- and instructor-independent learning.

### Supplementary Information

**Additional file 1.** Some free-text comments from participants. Free-text responses were collected with an open response item. Comments of the participants generally indicated a good acceptance. However, critical aspects were illuminated as well.

## Data Availability

Data used in this study are available upon reasonable request from the corresponding author at the Emergency Department of the University Hospital Bern, Switzerland to researchers eligible under Swiss legislation to work with codified personal health care data. Eligibility will be determined by Cantonal Ethics Committee Bern.

## Abbreviations

ASQ: After-Scenario Questionnaire
IQR: Interquartile range
Med: Median
NASA-TLX: NASA-Task Load Index
REBOA: Resuscitative endovascular balloon occlusion of the aorta
SSQ: Simulator sickness questionnaire
SUS: System Usability Scale
USEQ: User Satisfaction Evaluation Questionnaire
VR: Virtual reality
