# Supplementary material for: Development and usability testing of a fully immersive VR simulation for REBOA training
Source: Int J Emerg Med. 2023 Oct 6;16:67. doi: 10.1186/s12245-023-00545-6 (PMC10559413; doi:10.1186/s12245-023-00545-6)
Supplement: Supplementary file 1 — Additional file 1. Some free-text comments from participants. Free-text responses were collected with an open response item. Comments of the participants generally indicated a good acceptance. However, critical aspects were illuminated as well. [file 12245_2023_545_MOESM1_ESM.docx]

# **Supplement 1**

## **Some free-text comments from participants:**

"Great way to practice courses of action or scenarios."

"I thought the VR experience was great and was able to get a good look into the REBOA catheter procedure"

"Interesting experience to have a VR headset on my head for once. But for me this is no substitute for other means of education, as there is too much support needed. Cost/benefit ratio is not right for me."

"Fun to learn with new technical tools. For me it was a very hands-on training."

"Sonography and X-ray are not as clear/obvious. For example, there were problems in introducing the guidewire because the sonography probe could not be moved to the insertion site. Hand changes for activities or fixations with the second hand are not as clearly evident in the simulation. In real life, however, they are."

"The system had some stickers in parts. Basically a good tool to train and gain insight into something."

"Technical aspects (e.g. blocking with what volume, what lumens to flush, how far to insert (landmarks for location) are not illuminated."

"Effort and success have an unfavourable relationship"

"I have never done such a simulation and have not inserted a REBOA. Therefore, I have no experience and saw this as an interesting exercise"

"Good exercise. I was able to empathize well with the situation."

"It was fun to practice with a VR simulator for once. However, I still don't really know how the REBOA works and how you would really introduce it. The hand movements aren't real and you also don't see where the catheter is ultimately placed."
